# Combination Treatment of Timosaponin BII and Pirfenidone Attenuated Pulmonary Fibrosis Through Anti-Inflammatory and Anti-Fibrotic Process in Rodent Pulmonary Fibrosis Model and Cellular Epithelial–Mesenchymal Transition Model

**DOI:** 10.3390/molecules30081821

**Published:** 2025-04-18

**Authors:** Xuebin Shen, Yueyue Zheng, Hui Yang, Li Liu, Lizhen Yu, Yuanxiang Zhang, Xiaojun Song, Yuqing He, Runze Jin, Jianhao Jiao, Zhihui Gu, Kefeng Zhai, Sihui Nian, Limin Liu

**Affiliations:** 1 School of Pharmacy, Wannan Medical College, Wuhu 241002, China; sxbchn@wnmc.edu.cn (X.S.); 20219062@stu.wnmc.edu.cn (Y.Z.); 20229101@stu.wnmc.edu.cn (H.Y.); liulilili@stu.wnmc.edu.cn (L.L.); yulizhen@wnmc.edu.cn (L.Y.); 20210027@wnmc.edu.cn (Y.Z.); tsongxj@wnmc.edu.cn (X.S.); 20229073@stu.wnmc.edu.cn (Y.H.); 20229114@stu.wnmc.edu.cn (R.J.); 20239138@stu.wnmc.edu.cn (J.J.); 20239128@stu.wnmc.edu.cn (Z.G.); 2 School of Pharmacy, Anhui College of Traditional Chinese Medicine, Wuhu 241003, China; 3 School of Biological and Food Engineering, Engineering Research Center for Development and High Value Utilization of Genuine Medicinal Materials in North Anhui Province, Suzhou University, Suzhou 234000, China; 4 Center for Xin’an Medicine and Modernization of Traditional Chinese Medicine of IHM, Hefei 230051, China

**Keywords:** timosaponin BII, pirfenidone, combination treatment, anti-inflammatory, anti-fibrotic, epithelial–mesenchymal transition, rodent PF model, cellular EMT model

## Abstract

Pulmonary fibrosis (PF) is a progressive lung disease with a poor prognosis. Pirfenidone (PFD) can slow down the decline of lung function, but defects in efficacy and accompanying side effects limit its application; hence, implementing methods including combination therapy might be a viable option. Given this, we hypothesized that combining timosaponin BII (TS BII) with PFD might offer a more effective treatment approach. Bleomycin-induced rodent PF model and TGF-β1-induced cellular epithelial–mesenchymal transition (EMT) model were applied in the study. The results showed that the combination of TS BII and PFD was more effective in reducing the production of IL-1β, TNF-α, collagen fibers, hydroxyproline, and MDA. Moreover, the combination treatment could better restore levels SOD and GSH-Px. In addition, TS BII combined with PFD could downregulate the expression of NF-κB and the ratio of p-IκBα/IκBα, and modulate the aberrant expression of epithelial–mesenchymal transition markers. In addition, the combination treatment could regulate the intestinal flora of PF mice. It is worth noting that among the above results, there were significant differences (*p* < 0.05) between the combination group and either the TS BII or PFD monotherapy group. These findings indicate that the combination of TS BII and PFD has a synergistic effect in the treatment of PF and represents a promising treatment strategy.

## 1. Introduction

Pulmonary fibrosis (PF) is an irreversible and progressive lung disorder, which is a peculiar kind of chronic fibrotic interstitial pneumonia that mainly occurs in the victim’s lungs [1,2]. PF is characterized by the proliferation of fibroblasts and secretion of abnormal extracellular matrix (ECM), which ultimately results in the lethal deterioration of the structure of normal lung tissue. The pathogenic mechanism of PF remains to be fully illustrated; however, with the progress of cellular and molecular biology in recent years, understanding of the physiological and pathological processes related to PF has been greatly enhanced. It is now widely accepted that inflammation, redox imbalance, and epithelial–mesenchymal transition (EMT) are critical during the initiation and progression of PF [3,4,5]. A variety of risk factors can induce PF, such as environmental and genetic factors, epigenetic alterations, fibroblastic foci and epithelium–mesenchyma crosstalk, inflammation and immunity, etc. [6]. During 2013–2018, it was indicated that the age- and sex-standardized mortality rate stood at 3.9 per 100,000 person-years. Even with the availability of approved anti-fibrotic treatments, the mortality rates associated with PF in Europe increase on average by 1.7% each year, and it is conservatively estimated that 17,000 individuals die from PF annually [7].

Pirfenidone (PFD), a pleiotropic pyridine compound, has shown the ability to combat fibrosis by suppressing inflammatory response and oxidative stress response [8]. It is one of the two drugs officially recommended for the treatment of PF [9]. However, PFD can only improve pulmonary function to a limited extent and patients often remain with poor lung function after medication treatment. In addition, it is frequently accompanied by unexpected side effects, such as cardiovascular risks and bleeding risks [10]. There is still a lack of highly effective clinical options for PF, and the existing therapeutic treatments can only delay the progression of the disease rather than offering a complete cure. In addition to medical treatment, lung transplantation can also provide patients with a certain elongation of lifespan. However, it is far from realistic for most patients to undergo lung transplantation due to high costs and a shortage of suitable donor organs. Therefore, it is of great significance to develop new and reliable therapeutic approaches for PF.

Traditional Chinese medicine (TCM) has long been used in disease treatment, and it has been reported that multiple total extracts or a single active component of TCM could attenuate PF. Their underlying mechanisms include reducing inflammation, modulating oxidative stress, regulating the immune system, and promoting tissue repair [11,12,13]. In recent years, a large number of clinical trials have attempted to treat PF with various TCM interventions alone or in combination with Western medicinal measures, and combination therapy has emerged as an attractive and promising approach [14].

*Anemarrhena asphodeloides* Bunge (AAB) is rich in multiple chemical components with diverse biological activities, and has been widely used in traditional medicine [15]. Timosaponin BII (TS BII) is one of the main active ingredients of AAB, and our previous work has confirmed that TS BII could impede abnormal collagen secretion, transforming growth factor-β (TGF-β)/Smad pathway initiation, and EMT in both in vivo and in vitro models [16]. Considering the limitations of PFD and the potential of TS BII, we hypothesized that there might be a synergistic effect between TS BII and PFD in the treatment of PF.

In this study, we aimed to investigate the protective effects of TS BII and PFD, either alone or in combination, against PF. We used a bleomycin (BLM)-induced mouse PF model and a TGF-β1-induced EMT cell model to comprehensively explore the impact of this combination strategy on PF, with the hope of providing new treatment ideas and options for PF patients.

## 2. Results

### 2.1. TS BII, PFD, and Their Combination Exerted Anti-Inflammatory Effects

**Figure 1:**
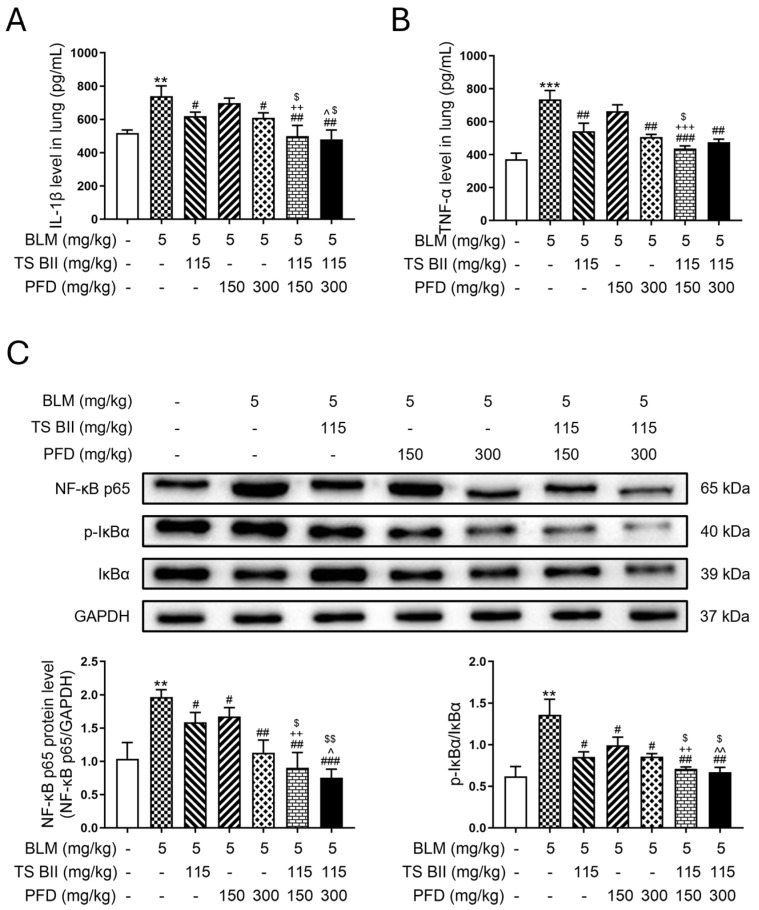
Effects of TS BII and/or PFD on inflammatory cytokines in vivo. (**A**,**B**) represent levels of interleukin-1β (IL-1β) and tumor necrosis factor-α (TNF-α), respectively. (**C**) represents the Western blot results of nuclear factor-κB (NF-κB) and ratio of p-IκBα/IκBα. ** *p* < 0.01 vs. control group; *** *p* < 0.001 vs. control group; # *p* < 0.05 vs. model group; ## *p* < 0.01 vs. model group; ### *p* < 0.001 vs. model group; ++ *p* < 0.01 vs. PFD (150 mg/kg) group; +++ *p* < 0.001 vs. PFD (150 mg/kg) group; ^ *p* < 0.05 vs. PFD (300 mg/kg) group; ^^ *p* < 0.01 vs. PFD (300 mg/kg) group; $ *p* < 0.05 vs. TS BII group; $$ *p* < 0.01 vs. TS BII group (*n* = 3).

BLM induction led to a marked elevation of interleukin-1β (IL-1β) (Figure 1A) and tumor necrosis factor-α (TNF-α) (Figure 1B) in the lungs of PF mice. Single treatments with TS BII or PFD could reverse this increase. However, the combination of TS BII and PFD demonstrated a more significant reduction in the level of IL-1β compared to single-treatment groups. Moreover, Western blot results indicated that in the model group, the expression of nuclear factor-κB (NF-κB) and the ratio of p-IκBα/IκBα were augmented (Figure 1C). This abnormal increase was reversed by either single treatments or a combination treatment. Notably, the combination groups showed a greater decrease in the expression of NF-κB and the p-IκBα/IκBα ratio compared to single TS BII or PFD treatment groups.

**Figure 2:**
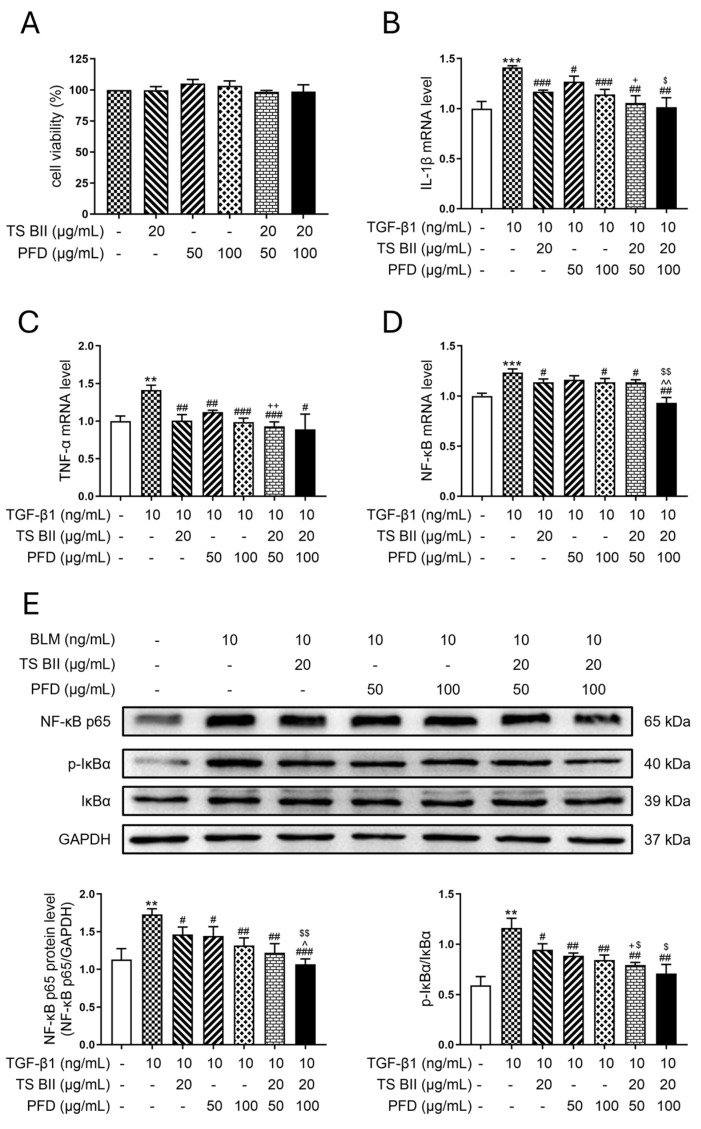
Effects of TS BII and/or PFD on inflammatory cytokines in vitro. (**A**) represents cell viability upon TS BII and/or PFD administration. (**B**–**D**) represent mRNA levels of *interleukin-1β (IL-1β), tumor necrosis factor-α (TNF-α)*, and *nuclear factor-κB (NF-κB)*, respectively. (**E**) represents the Western blot results of nuclear factor-κB (NF-κB) and the ratio of p-IκBα/IκBα. ** *p* < 0.01 vs. control group; *** *p* < 0.001 vs. control group; # *p* < 0.05 vs. model group; ## *p* < 0.01 vs. model group; ### *p* < 0.001 vs. model group; + *p* < 0.05 vs. PFD (150 mg/kg) group; ++ *p* < 0.01 vs. PFD (150 mg/kg) group; ^ *p* < 0.05 vs. PFD (300 mg/kg) group; ^^ *p* < 0.01 vs. PFD (300 mg/kg) group; $ *p* < 0.05 vs. TS BII group; $$ *p* < 0.01 vs. TS BII group (*n* = 3).

In vitro cytotoxicity of TS BII and/or PFD on MRC-5 cells indicated that TS BII at a concentration of 20 μg/mL, and PFD at concentrations of 50 μg/mL and 100 μg/mL, whether used alone or in combination, did not exhibit cytotoxicity when acting on MRC-5 cells for 24 h (Figure 2A). Further study depicted that in the in vitro model, after the TGF-β1 administration to MRC-5 cells, the mRNA expression of *IL-1β* (Figure 2B), *TNF-α* (Figure 2C), and *NF-κB* (Figure 2D) increased. Western blotting also revealed an upregulation of NF-κB expression and an amplification of the p-IκBα/IκBα ratio in the TGF-β1 group. Treatments with TS BII or PFD, either alone or in combination, reversed the aberrant expression of these inflammatory cytokines induced by TGF-β1 (Figure 2E). The combination treatment showed a more prominent anti-inflammatory effect, with significant differences compared to single-treatment groups.

### 2.2. TS BII, PFD, and Their Combination Exerted Anti-Fibrotic Effects

**Figure 3:**
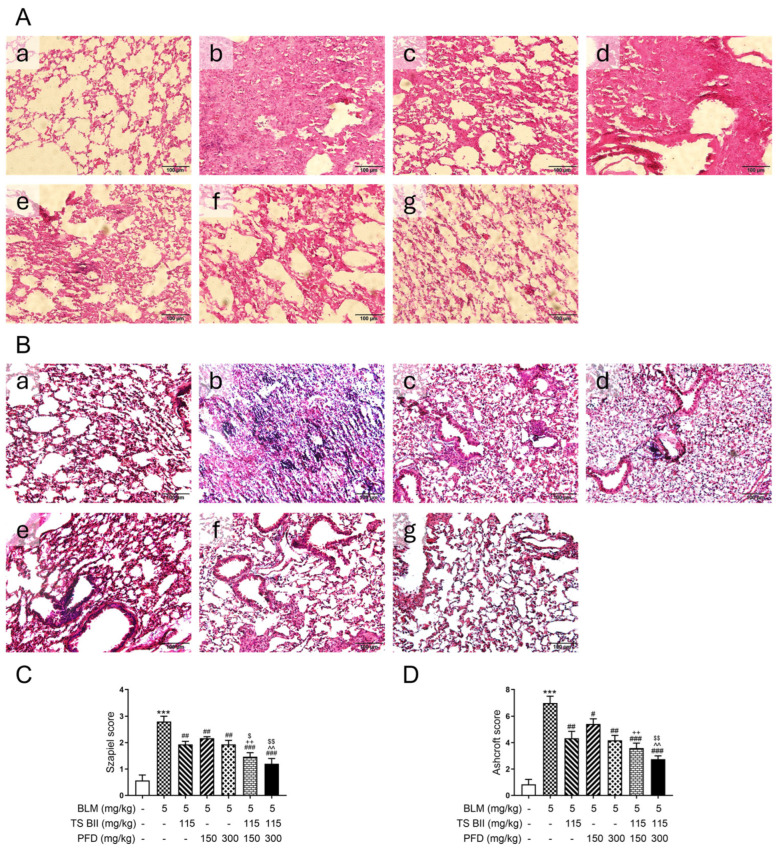
Histopathological changes in lung tissues in vivo. H&E staining demonstrated the lung histopathological abnormalities (**A**) and Masson’s trichrome staining indicated collagen deposition (**B**). (**C**) represents the Szapiel score and (**D**) represents the Ashcroft score. In this figure, (**a**) control group, (**b**) model group, (**c**) TS BII group, (**d**) PFD (150 mg/kg) group, (**e**) PFD (300 mg/kg) group, (**f**) TS BII + PFD (150 mg/kg) group, and (**g**) TS BII + PFD (300 mg/kg) group. The scale bar represents 100 μm, and all images were taken at a magnification of 200×. *** *p* < 0.001 vs. control group; # *p* < 0.05 vs. model group; ## *p* < 0.01 vs. model group; ### *p* < 0.001 vs. model group; ++ *p* < 0.01 vs. PFD (150 mg/kg) group; ^^ *p* < 0.01 vs. PFD (300 mg/kg) group; $ *p* < 0.05 vs. TS BII group; $$ *p* < 0.01 vs. TS BII group (*n* = 3).

In the histopathological examination by H&E staining (Figure 3A), the alveolar structure in the control group was intact, while the model group showed a thickened alveolar septum. TS BII and two doses of PFD treatment could decrease the alveolar wall thickness and increase the alveolar space. The combination treatment had a more obvious amelioration in the structural destruction of mouse lungs. Moreover, Masson’s staining (Figure 3B) indicated that the model group had an extensive blue-stained area in the lung tissue, representing severe PF. The TS BII and/or PFD treatment reduced this area. The protective effect of PFD was enhanced by increasing the dosage or combining it with TS BII. In addition, the Szapiel (Figure 3C) and Ashcroft (Figure 3D) scores also demonstrated that BLM-induced alveolitis and fibrosis were alleviated by TS BII and/or PFD administration. The combination of PFD and TS BII had a better anti-fibrotic effect than single treatments.

**Figure 4:**
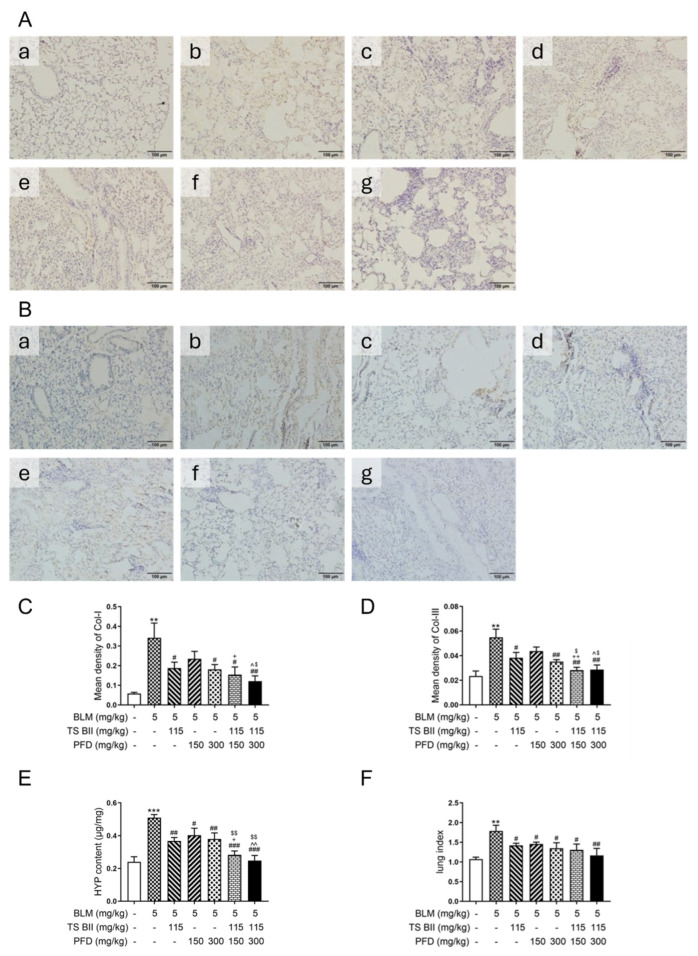
Expression of collagen type I (Col-I), collagen type III (Col-III), hydroxyproline (HYP) content, and lung index in vivo. Col-I (**A**,**C**) and Col-III (**B**,**D**) deposition were examined by an immunohistochemistry (IHC) analysis. HYP (**E**) level and lung index (**F**) were detected. In this figure, (**a**) control group, (**b**) model group, (**c**) TS BII group, (**d**) PFD (150 mg/kg) group, (**e**) PFD (300 mg/kg) group, (**f**) TS BII + PFD (150 mg/kg) group, and (**g**) TS BII + PFD (300 mg/kg) group. The scale bar represents 100 μm, and all the images were taken at a magnification of 200×. ** *p* < 0.01 vs. control group; *** *p* < 0.001 vs. control group; # *p* < 0.05 vs. model group; ## *p* < 0.01 vs. model group; ### *p* < 0.001 vs. model group; + *p* < 0.05 vs. PFD (150 mg/kg) group; ++ *p* < 0.01 vs. PFD (150 mg/kg) group; ^ *p* < 0.05 vs. PFD (300 mg/kg) group; ^^ *p* < 0.01 vs. PFD (300 mg/kg) group; $ *p* < 0.05 vs. TS BII group; $$ *p* < 0.01 vs. TS BII group (*n* = 3).

In terms of collagen production, BLM stimulation increased the expression levels of collagen type I (Col-I) (Figure 4A,C) and collagen type III (Col-III) (Figure 4B,D), as well as the hydroxyproline (HYP) content (Figure 4E). Treatments with TS BII or PFD reversed these increases. The combination of TS BII and PFD had a more significant positive effect on reducing collagen production compared to the PFD-only group. Moreover, the lung index score (Figure 4F), which was significantly increased in the model group, was decreased in the groups treated with TS BII and/or PFD, and the combination treatment was more effective.

### 2.3. TS BII, PFD, and Their Combination Modulated Oxidative Stress

**Figure 5:**
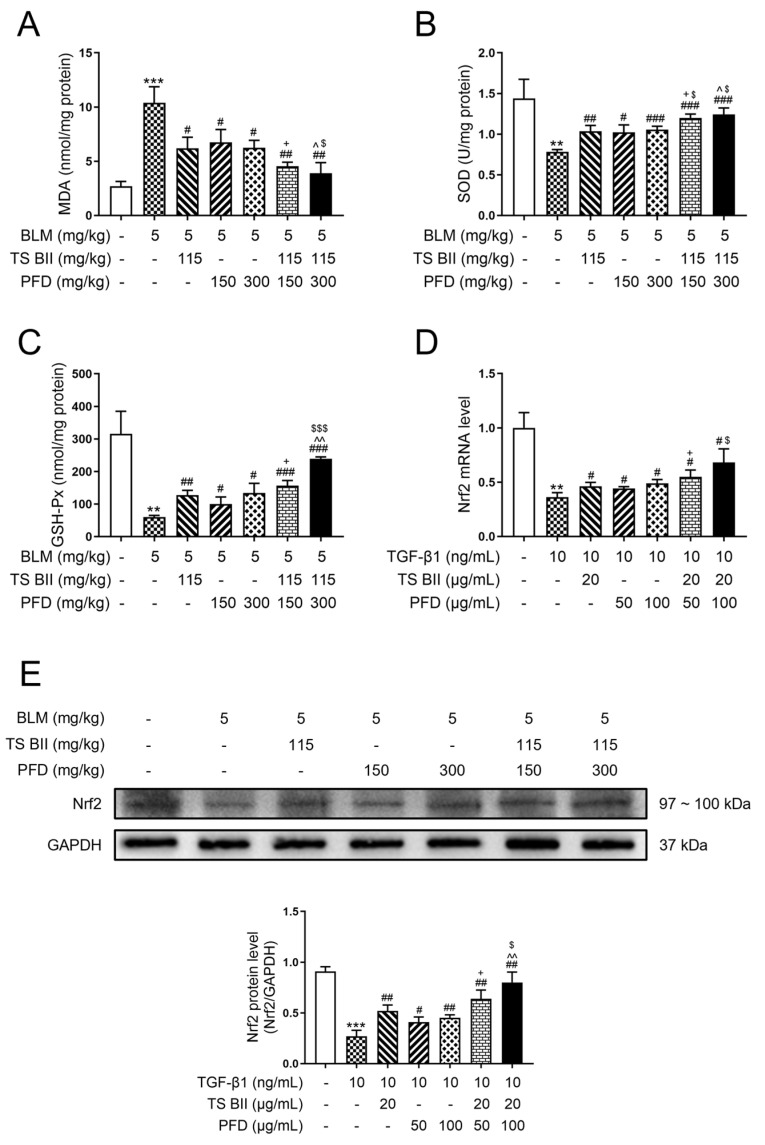
Effect of TS BII and/or PFD on oxidative stress in vivo and in vitro. (**A**–**C**) represent the results of oxidant indicator of malondialdehyde (MDA), antioxidant indicators super-oxide dismutase (SOD), and glutathione peroxidase (GSH-Px), respectively. (**D**) represents the qRT-PCR result of nuclear factor erythroid 2-related factor 2 (Nrf2) in vitro, and (**E**) represents the Western blot result of Nrf2 in vivo. ** *p* < 0.01 vs. control group; *** *p* < 0.001 vs. control group; # *p* < 0.05 vs. model group; ## *p* < 0.01 vs. model group; ### *p* < 0.001 vs. model group; + *p* < 0.05 vs. PFD (150 mg/kg) group; ^ *p* < 0.05 vs. PFD (300 mg/kg) group; ^^ *p* < 0.01 vs. PFD (300 mg/kg) group; $ *p* < 0.05 vs. TS BII group; $$$ *p* < 0.001 vs. TS BII group (*n* = 3).

As can be seen in Figure 5A, lipid oxidation indicator malondialdehyde (MDA) was significantly increased, while the levels of antioxidant substances such as superoxide dismutase (SOD) (Figure 5B) and glutathione peroxidase (GSH-Px) (Figure 5C) were drastically reduced under BLM intoxication. Interestingly, TS BII, PFD, and their combination significantly increased the SOD and GSH-Px levels and decreased the MDA level, while the combination treatments showed a better effect compared to single-treated groups.

In addition, the qRT-PCR detection of nuclear factor erythroid 2-related factor 2 (Nrf2) mRNA (Figure 5D) in MRC-5 cells and Western blot detection of Nrf2 protein (Figure 5E) in the rodent model showed that the expression of Nrf2 was downregulated after the BLM or TGF-β1 administration. Both combination treatments could significantly restore Nrf2 expression compared to the model group, with significant differences compared to the monotherapy groups.

### 2.4. TS BII, PFD, and Their Combination Regulated Epithelial–Mesenchymal Transition (EMT)

**Figure 6:**
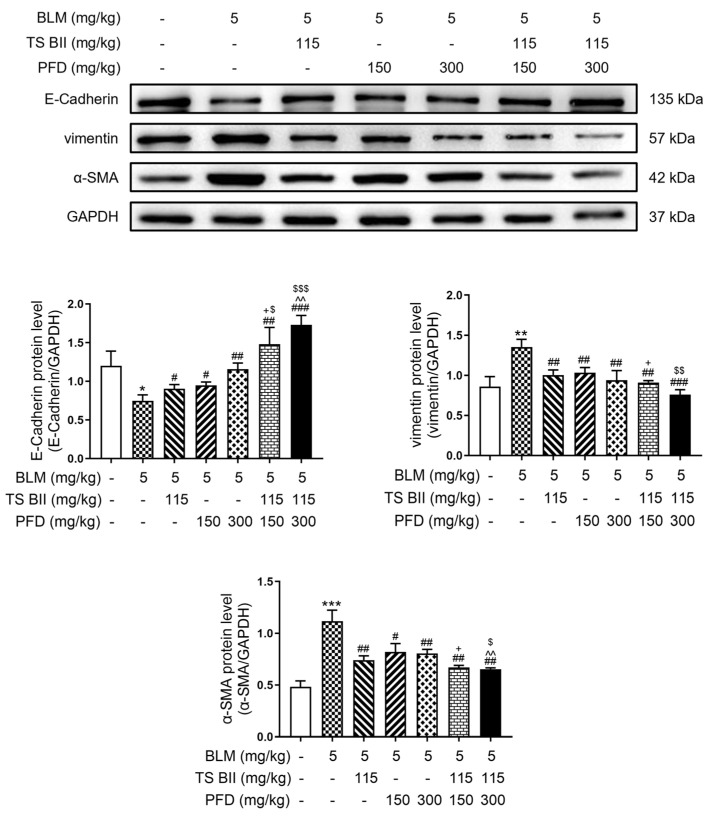
Effect of TS BII and/or PFD on EMT-related indicators in vivo. * *p* < 0.05 vs. control group; ** *p* < 0.01 vs. control group; *** *p* < 0.001 vs. control group; # *p* < 0.05 vs. model group; ## *p* < 0.01 vs. model group; ### *p* < 0.001 vs. model group; + *p* < 0.05 vs. PFD (150 mg/kg) group; ^^ *p* < 0.01 vs. PFD (300 mg/kg) group; $ *p* < 0.05 vs. TS BII group; $$ *p* < 0.01 vs. TS BII group; $$$ *p* < 0.001 vs. TS BII group (*n* = 3).

As depicted in Figure 6, BLM induction downregulated the expression of E-cadherin and upregulated the expression of vimentin and α-SMA. Treatments with TS BII or PFD could significantly reverse these abnormal expressions of EMT-marker proteins. The differences between the single-treatment groups and the combined-treatment groups were statistically significant, indicating that the combination treatment was more effective in modulating BLM-induced EMT in vivo.

**Figure 7:**
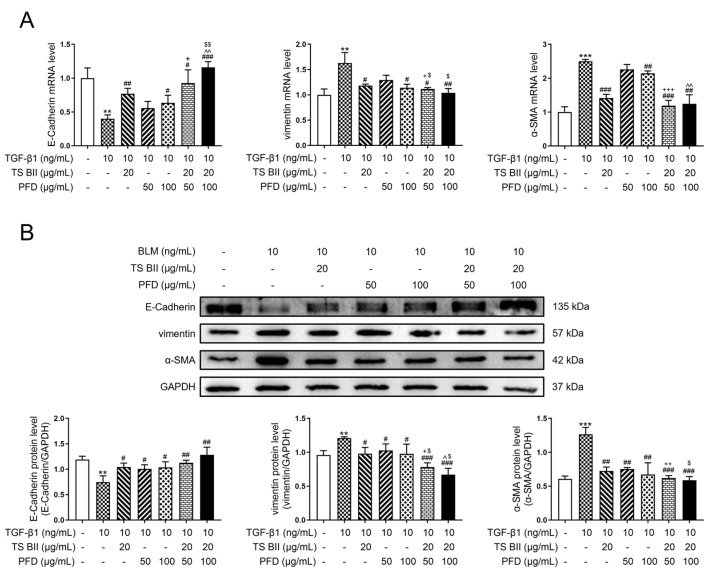
Effect of TS BII and/or PFD on EMT-related indicators in vitro. Representative results of the EMT-related indicators (E-cadherin, vimentin, and α-SMA) detected by the qRT-PCR (**A**) and Western blot (**B**) analysis. ** *p* < 0.01 vs. control group; *** *p* < 0.001 vs. control group; # *p* < 0.05 vs. model group; ## *p* < 0.01 vs. model group; ### *p* < 0.001 vs. model group; + *p* < 0.05 vs. PFD (150 mg/kg) group; ++ *p* < 0.01 vs. PFD (150 mg/kg) group; +++ *p* < 0.001 vs. PFD (150 mg/kg) group; ^ *p* < 0.05 vs. PFD (300 mg/kg) group; ^^ *p* < 0.01 vs. PFD (300 mg/kg) group; $ *p* < 0.05 vs. TS BII group; $$ *p* < 0.01 vs. TS BII group (*n* = 3).

**Figure 8:**
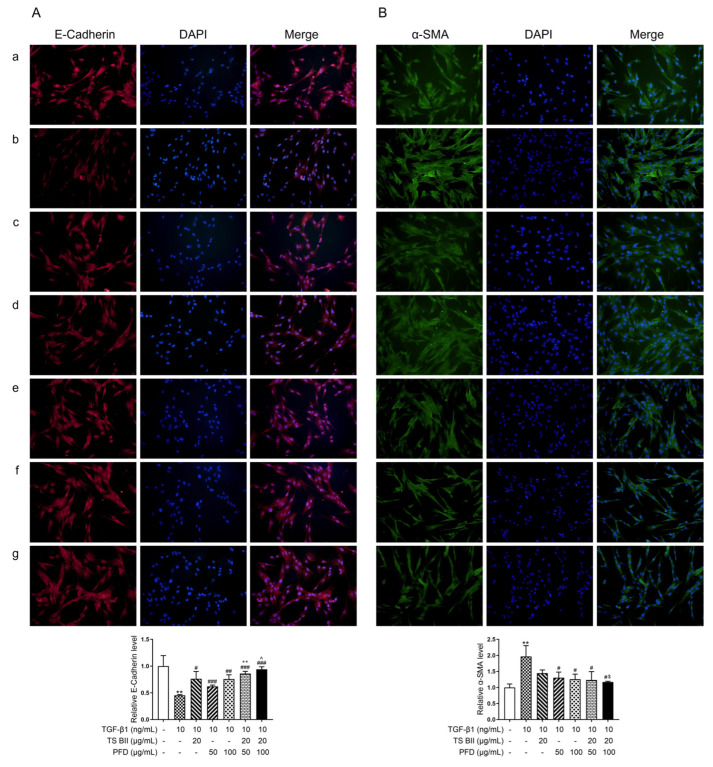
Immunofluorescence (IF) staining results of E-cadherin (**A**) and α-SMA (**B**) expression in MRC-5 cells. In this figure, (**a**) control group, (**b**) model group, (**c**) TS BII group, (**d**) PFD (150 mg/kg) group, (**e**) PFD (300 mg/kg) group, (**f**) TS BII + PFD (150 mg/kg) group, and (**g**) TS BII + PFD (300 mg/kg) group. ** *p* < 0.01 vs. control group; # *p* < 0.05 vs. model group; ## *p* < 0.01 vs. model group; ### *p* < 0.001 vs. model group; ++ *p* < 0.01 vs. PFD (150 mg/kg) group; ^ *p* < 0.05 vs. PFD (300 mg/kg) group; $ *p* < 0.05 vs. TS BII group (*n* = 3).

In the in vitro exploration, Figure 7A,B indicated that the TGF-β1 administration significantly decreased the epithelial marker E-cadherin mRNA level and increased the mesenchymal markers vimentin and α-SMA mRNA levels in MRC-5 cells. Except for the single treatment with 50 μg/mL PFD, TS BII, PFD (100 μg/mL), and their combination could significantly reverse this fibrotic effect. The combination treatment further enhanced the protective effect, showing a significant difference compared to single-treatment groups. In addition, the immunofluorescence (Figure 8A,B) staining results also indicated that the combination treatment was more effective in alleviating the abnormal EMT process related to fibrosis.

### 2.5. TS BII, PFD, and Their Combination Regulated Intestinal Flora

**Figure 9:**
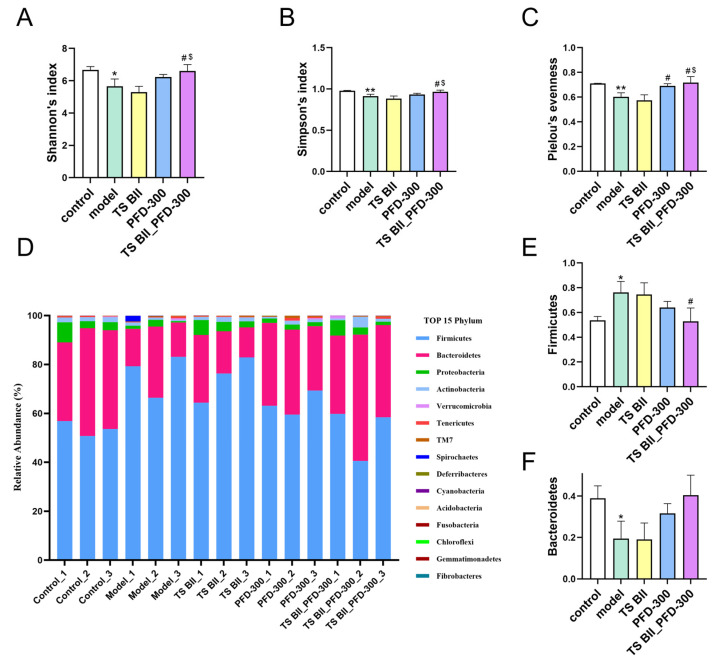
Effect of TS BII and/or PFD (300 mg/kg) on intestinal flora in vivo. Shannon’s (**A**), Simpson’s (**B**), and Pielou’s (**C**) evenness indexes. Changes in the top 20 strains (**D**), Firmicutes (**E**), and Bacteroidetes (**F**) in the intestinal flora at the phylum level. * *p* < 0.05 vs. control group; ** *p* < 0.01 vs. control group; # *p* < 0.05 vs. model group; $ *p* < 0.05 vs. TS BII group (*n* = 3).

As depicted in Figure 9A–C, Shannon’s, Simpson’s, and Pielou’s evenness indexes were all lowered due to BLM induction, yet this toxic reaction was reversed by the combination therapy of TS BII combined with 300 mg/kg PFD. Further analysis showed that the distribution of microflora in mice at the phylum level was affected by PF (Figure 9D–F). The availability of TS BII and 300 mg/kg PFD administration could vary this effect. For example, the relative abundance of Firmicutes and Bacteroidetes increased and decreased, respectively, under BLM stimulation, and the combination of TS BII and PFD could regulate this alteration.

## 3. Discussion

PF is a devastating lung disorder with complex pathogenesis and limited treatment options. PFD, an FDA-approved drug for PF, has shown some efficacy in retarding disease progression [1,8]. However, its effectiveness is hampered by side effects and incomplete symptom alleviation [10,17]. Our previous research indicated that TS BII, a component of AAB, could alleviate PF [16]. This study aimed to explore the potential synergistic effect of combining TS BII and PFD in treating PF.

Inflammatory cytokines play a pivotal role in the pathogenesis of PF. In the resting state, NF-κB is located in the cytoplasm and remains inactive due to the formation of a polymer with its inhibitor IκBα. IκBα is phosphorylated upon stimulation and, in turn, provokes NF-κB to translocate from cytoplasm to nucleus, thus mobilizing the release of inflammatory cytokines and leading to inflammation [18,19,20]. Therefore, the level of NF-κB as well as the ratio of p-IκBα to IκBα are closely correlated with the severity of the inflammatory response. In addition, the overexpression of IL-1β in the lung can result in the elevation of pro-inflammatory cytokines including TNF-α, leading to severe lung injury [21]. Noteworthily, TNF-α is one of the cytokines that display strongly responsive to pro-inflammatory stimuli, inducing the production of other cytokines and mediating leukocyte chemotaxis. In addition, it is deciphered that TNF-α is involved in the pathogenesis of inflammatory diseases not only by potentiating inflammatory mediators expression but also by triggering cell death [22]. In this work, we examined the production of inflammatory factors in both mice and cell models, and the results suggested that compared with the control group, TNF-α, IL-1β, and NF-κB were significantly increased, and the ratio of p-IκBα to IκBα was also remarkably augmented. However, these effects in the model group were reversed in TS BII and/or PFD administration groups. Notably, the combination of TS BII and PFD showed a substantially lowering effect than a single treatment, suggesting a synergistic effect of these two reagents.

The results of this study clearly demonstrated a significant synergistic effect between TS BII and PFD. In terms of histopathological changes, both drugs, when used in combination, exerted a more remarkable effect on reducing alveolar wall thickening and collagen deposition compared to single-drug treatments. This was evident from the decreased, blue-stained area in Masson’s staining and reduced the Szapiel and Ashcroft scores, suggesting a more effective inhibition of fibrosis progression. Moreover, collagen production is a hallmark of PF [23,24]. The combined treatment of TS BII and PFD led to a more significant reduction in the expression levels of Col-I and Col-III, as well as HYP content, compared to individual drug treatments. This indicates that the combination can more potently inhibit the excessive production of extracellular matrix components, thereby alleviating the fibrotic process.

Redox imbalance is a crucial factor in PF development. MDA is the major product of lipid peroxidation which acts as a biomarker of oxidative stress and is malicious to cells and tissues due to its severe toxicity [25]. On the other hand, antioxidant enzymes such as SOD and GSH-Px are pivotal in coping with the dramatic augmentation in free radical production through scavenging free radicals [26]. The balance of oxidants and antioxidants is destroyed during PF which subsequently leads to damage. Dysregulated increment of oxidants and decrement of antioxidants were found in model animals in this work, but treatment with either TS BII or PFD could retrieve the antioxidant capacity and reduce the level of oxidant. For a profound illustration of the anti-oxidative effect of TS BII and/or PFD, the upstream regulator Nrf2 was explored. Nrf2 is a main factor that responds to changes in redox state and serves as a primary antioxidants regulator by activating antioxidant response elements (AREs) [27,28]. In response to oxidative stress, Nrf2 promotes antioxidant capacity by translocating to the nucleus and binding to AREs [29]. Papers have proved that Nrf2 shows a protective role in various lung diseases by reducing oxidative stress [30,31]. Consistently, single treatment with TS BII or PFD could promote the release of Nrf2. In addition, the combined treatment groups showed a better effect in this work, suggesting that TS BII combined with PFD could enhance the antioxidant capacity compared with the monotherapy groups.

It has been reported that the intestinal flora could be affected by PF [32]; hence, we hoped to gain a brief understanding of the gut microbiota diversity and evenness of the PF models in our study. Shannon’s, Simpson’s and Pielou’s diversity indexes are indicators that represent the richness and evenness of microbiota species [33]. In this study, both the richness and evenness of the intestinal flora were altered in the model group, and this effect was reversed by a combination of TS BII and PFD. The changes in Firmicutes and Bacteroidetes in the model group could also be modulated by combination treatment with TS BII and PFD. Although the exact mechanism remains to be further investigated, this finding implied a potential additional benefit of the combination treatment in modulating the gut–lung axis.

However, it is of great importance to note that TS BII belongs to saponins, which are plant compounds with potential toxicity. In the context of PF treatment, which often requires long-term or permanent administration, this poses significant concerns. The results of our cytotoxicity evaluation revealed that within the scope of this study, neither the sole application of TS BII nor its combined use with PFD demonstrated any cytotoxicity towards MRC-5 cells after 24 h treatment. However, the lack of long-term in vivo toxicity data of TS BII restricts our understanding of its safety profile during long-term use. Further research is urgently needed to explore strategies to mitigate these potential negative effects, such as optimizing the dosing regimen or developing targeted delivery systems.

## 4. Materials and Methods

### 4.1. Material and Reagents

Pentobarbital sodium was supplied by Sigma (St. Louis, MO, USA). Bleomycin (BLM) was obtained from Hanhui Pharmaceutical Co., Ltd. (Lot: 20026111, Shanghai, China). PFD was purchased from Beijing Continent Pharmaceuticals Co., Ltd. (Lot: 20220110, Beijing, China). The MTT, glutathione peroxidase, hydroxyproline, superoxide dismutase, and malondialdehyde assay kits were bought from Nanjing Jiancheng Bioengineering Institute (Nanjing, China). The IHC analysis kit was bought from Maxim Biotechnologies (Fuzhou, China). The Mouse IL-1β, IL-6, and TNF-α ELISA kits were supplied by ABclonal Technology Co., Ltd. (Wuhan, China). Hematoxylin and eosin (H&E) and Masson’s trichrome staining reagents were supplied by Beijing Regen Biotechnology Co., Ltd. (Beijing, China). The mouse monoclonal primary antibodies against Col-I and Col-III were purchased from Boster Biological Technology (Pleasanton, CA, USA). DAB reagent and ultrasensitive SP kit were purchased from Maxim Biotechnologies (Fuzhou, China). Primary antibodies against E-cadherin, vimentin, α-SMA, p-IκBα, IκBα, NF-κB, and Nrf2 were purchased from CST (Danvers, MA, USA). GAPDH primary antibody, goat anti-rabbit secondary antibody, and qRT-PCR kit were supplied by Beijing Labgic Technology (Beijing, China). The Trizol and RevertAid First Strand cDNA synthesis kits were purchased from Thermo Fisher Scientific (Waltham, MA, USA). For IF staining, Rabbit polyclonal antibodies against E-cadherin and α-SMA were supplied by Affinity Biosciences (Changzhou, China), goat anti-rabbit secondary antibody was purchased from Boster Biological Technology (Pleasanton, CA, USA), goat serum for blocking and DAPI were bought from Beijing Labgic Technology (Beijing, China).

AAB was obtained from the Bozhou Chinese Herbal Medicinal Market and was identified by Professor Shuyun Bao (School of Pharmacy, Wannan Medical College). The voucher specimen was preserved at Wannan Medical College, Anhui Province, China. For the purification of TS BII [34], dried AAB was crushed into granules, then extracted with 70% ethanol, and purified by column chromatography with macroporous resin HPD400. The purity of TS BII was identified by HPLC and the purity is about 98%.

### 4.2. Animals

Healthy adult male C57BL/6J mice (18–22 g) (Certificate No. SCXK[Yu]2020-0005) were bought from He’nan Skbex Biotechnology Co., Ltd. (Anyang, China). The mice were maintained under the temperature of 22–26 °C with a light/dark cycle of 12 h, respectively, and free access to water and food was allowed during the research. Mice were domesticated for 7 days, then drugs were administered before fasting for 12 h. The protocol of this work was authorized by the Animal Ethics Committee of Wannan Medical College, and all procedures were performed according to the Guidelines for Animal Experiments of Wannan Medical College (Ethics permit number: WNMC-AWE-2023468).

### 4.3. Establishment of Mouse PF Model and Drug Administration

A total of 42 healthy male C57BL/6J mice were randomly assigned into 7 groups: (a) control group (treated with normal saline), (b) model group (treated with 5 mg/kg BLM), (c) TS BII group (treated with 5 mg/kg BLM and 115 mg/kg TS BII), (d) PFD group (treated with 5 mg/kg BLM and 150 mg/kg PFD), (e) PFD group (treated with 5 mg/kg BLM and 300 mg/kg PFD), (f) TS BII + PFD group (treated with 5 mg/kg BLM, 115 mg/kg TS BII and 150 mg/kg PFD), and (g) TS BII + PFD group (treated with 5 mg/kg BLM, 115 mg/kg TS BII and 300 mg/kg PFD). The mice were anesthetized with 40 mg/kg pentobarbital sodium by intraperitoneal injection. Except for the control group, the mice received an intratracheal infusion of BLM. TS BII and PFD were dissolved in 0.5% sodium carboxymethyl cellulose and administered intragastrically alone or in combination for 14 consecutive days.

### 4.4. Pulmonary Histopathological Examination

For lung index determination, all the mice were sacrificed when the drug administration was completed, and lung tissues were collected and weighed. Lung index score was determined by Formula (1):

(1) $$lung\;index=\frac{lung\;weight\;(mg)}{body\;weight\;(g)}$$

To investigate the BLM-induced fibrotic and histological abnormalities, a pulmonary histopathological examination was performed. Samples of mice lung tissue were perfused and fixed by 4% paraformaldehyde for 24 h. The tissues were then dehydrated with ethanol at different concentrations (75% for 1.5 h, 85% for 1.5 h, 95% for 1.5 h, 100% for 1 h twice) and hyalinized with xylene (50% xylene and 50% ethanol for 1 h, 100% xylene for 1 h twice). Lung specimens were then embedded in paraffin and sliced into 5 μm sections before being stained by agents of H&E or Masson, respectively, and the images were acquired by a light microscope.

### 4.5. Biochemical Parameters Measurement

The lung tissue was first homogenized with precooled normal saline before centrifuging at 5000 rpm for 15 min at 4 °C. The supernatants were then transferred for analysis of GSH-Px, HYP, IL-1β, SOD, and TNF-α with commercial kits, and assays were carried out in accordance with the procedures provided by the manufacturers.

### 4.6. IHC Analysis

The lung slices embedded with paraffin were treated with specific reagents for the expression determination of Col-I and Col-III levels by IHC analysis. The sections were dewaxed and treated with gradient ethanol and xylene before being heated in citric acid. Then, the samples were treated with Col-I and Col-III antibodies at a dilution ratio of 1:200. DAB reagent was then used for positive staining, and hematoxylin was applied for nuclei staining. Stained sections were visualized by a light microscope and analyzed by ImageJ.

### 4.7. Cell Culture and Drug Administration

The human embryonic lung fibroblast MRC-5 cell line was supplied by Procell Life Science & Technology Co., Ltd. (Wuhan, China), and the passage number of the cell line was authenticated by Procell. Cells were maintained in MEM supplemented with non-essential amino acid, 10% fetal bovine serum, 100 μg/mL streptomycin, and 100 U/mL penicillin in a 37 °C, humidified sterile incubator with 5% CO_2_. When the cells reached a confluence of 90%, they were treated with 0.5% trypsin-EDTA and seeded into plates. Prior to explorations in the in vitro model, an MTT kit was utilized to detect the cytotoxicity of TS BII and PFD when used alone and in combination with MRC-5 cells. The cells were treated with TS BII (20 μg/mL) and/or PFD (50, 100 μg/mL) for 24 h, and then the subsequent operations were carried out in accordance with the instructions provided by the kit manufacturer.

### 4.8. qRT-PCR Assay

The MRC-5 cells were induced by TGF-β1 (10 ng/mL) and incubated with or without TS BII and/or PFD for 24 h, and then mRNA was isolated from the cells, and the mRNA level of indicators was tested by qRT-PCR. The total RNA from the cells was isolated by TRIzol before being reverse-transcribed into cDNA by a specific kit. The qRT-PCR was performed according to the protocol described in Table 1.

The details of the primers used in the test were listed in Table 2 and the primers were synthesized by GENERAL BIOL (Chuzhou, China). mRNA level was calculated according to the 2^−ΔΔCt^ method [35] and GAPDH served as the internal control.

### 4.9. Western Blot Analysis

For detecting the expression levels of proteins in mice lung tissues and cells, a Western blot analysis was used in this work. Total proteins from the lung tissues and MRC-5 cells were harvested with RIPA lysate buffer with PMSF, protease inhibitor, and phosphatase inhibitor. Protein samples of the same amount were transferred onto nitrocellulose membranes subsequent to separation by SDS-PAGE. Then, the membranes were treated in 5% non-fat milk dissolved in TBST for 3 h at room temperature and incubated with NF-κB, p-IκBα, IκBα, Nrf2, E-cadherin, vimentin, and α-SMA antibodies overnight at 4 °C. The dilution ratio of the antibodies was 1:1000. Then, the membranes were treated with TBST for 10 min thrice, followed by incubating with a secondary antibody with a dilution ratio of 1:10,000 for 2 h at room temperature, and treating with TBST for 10 min thrice. The labeling of the protein bands was developed by commercial enhanced chemiluminescence reagents. Images were obtained by Tanon 5200 Multi (Tanon, Shanghai, China) and quantified by ImageJ (Version 1.53t, NIH, Bethesda, MD, USA). p-IκBα was normalized to IκBα, while the expression level of GAPDH served as the internal control of other proteins.

### 4.10. IF Staining

The expression levels of intracellular E-Cadherin and α-SMA were examined by IF staining. The MRC-5 cells were treated with phosphate-buffered saline (PBS) and fixed with 4% paraformaldehyde for 15 min at 4 °C. Then, the cells were treated with precooled PBS and Triton X-100 for 10 min. The samples were subsequently treated with PBS for 5 min thrice before blocking with goat serum for 30 min. Next, the cells were incubated with antibodies against E-cadherin and α-SMA at a dilution ratio of 1:500 at 4 °C overnight. After washing with PBS for 5 min thrice, cells were then incubated with a secondary antibody (1:200) for 1 h at room temperature, followed by nucleus labeling with DAPI. The fluorescence images were captured by microscope and analyzed with ImageJ (Version 1.53t, NIH, Bethesda, MD, USA).

### 4.11. Intestinal Flora Analysis in BLM-Induced PF Mice

In order to detect the changes in the intestinal flora in the mice with PF and after drug treatment, feces from the colon of the control, model, TS BII, PFD (300 mg/kg), and TS BII+ PFD (300 mg/kg) groups were collected from the sacrificed mice, and the samples were maintained in dry ice and sent to the commercial testing institute for analysis.

### 4.12. Statistical Analysis

Statistical analyses were performed by SPSS19.0 for Windows, and the results were expressed as $\bar{x}\;$ ± SD. All the tests were conducted with at least three replicates, and differences between two groups were conducted with a one-way analysis of variance, and *p* < 0.05 was considered statistically significant.

## 5. Conclusions

In conclusion, the combination of TS BII and PFD showed a remarkable synergistic effect in treating PF. It effectively reduces inflammation, counteracts collagen deposition, restores redox balance, inhibits EMT, and modulates the intestinal flora. These findings suggest that the combination of TS BII and PFD could be a promising treatment strategy for PF, providing new hope for patients suffering from this intractable disease. However, further studies are needed to fully elucidate the underlying mechanisms and evaluate the long-term safety and efficacy of this combination treatment.

## Figures and Tables

**Table 1:** qRT-PCR protocol.

Step	Temperature	Note
1	95 °C	
2	95 °C	repeat steps 2 and 3 for 40 cycles
3	60 °C	

**Table 2:** qRT-PCR primer sequences.

Gene	Forward Primer	Reverse Primer
*IL-1β*	ATGATGGCTTATTACAGTGGCAA	GTCGGAGATTCGTAGCTGGA
*TNF-α*	CCTGCTGCACTTTGGAGTGA	GAGGGTTTGCTACAACATGGG
*NF-κB*	TGTAAAACGACGGCCAGT	CAGGAAACAGCTATGACC
*Nrf2*	TCCGGGTGTGTTTGTTCCAA	CGCCCGCGAGATAAAGAGTT
*E-cadherin*	CGATTCAAAGTGGGCACAGATG	GTAGGTGGAGTCCCAGGCGTAG
*Vimentin*	TCTGGATTCACTCCCTCTGGTT	ATCGTGATGCTGAGAAGAGTCTC
*α-SMA*	ATGCTCCCAGGGCTGTTTTC	CTTTTGCTCTGTGCTTCGGTC
*GAPDH*	CTTTGGTATCGTGGAAGGACTC	GTAGAGGCAGGGATGATGTTCT

## Data Availability

All the data are contained within the article and Supplementary Materials.

## Supplementary Materials

The following supporting information can be downloaded at: https://www.mdpi.com/article/10.3390/molecules30081821/s1. File S1: Western Blot original images. File S2: Western Blot original images-GAPDH.

## Abbreviations

The following abbreviations are used in this manuscript:

AAB	*Anemarrhena asphodeloides* Bunge
AREs	antioxidant response elements
BLM	bleomycin
CMC-Na	sodium carboxymethyl cellulose
Col-I	collagen type I
Col-III	collagen type III
ECM	extracellular matrix
EMT	epithelial–mesenchymal transition
GSH-Px	glutathione peroxidase
H&E	hematoxylin and eosin
IHC	immunohistochemistry
HYP	hydroxyproline
IF	immunofluorescence
IL-1β	interleukin-1β
MDA	malondialdehyde
NF-κB	nuclear factor-κB
Nrf2	nuclear factor erythroid 2-related factor 2
PBS	phosphate-buffered saline
PF	pulmonary fibrosis
PFD	pirfenidone
qRT-PCR	quantitative real-time PCR
SOD	superoxide dismutase
TGF-β1	transforming growth factor-β1
TNF-α	tumor necrosis factor-α
TS BII	timosaponin BII
