# Supplementary material for: Combination Treatment of Timosaponin BII and Pirfenidone Attenuated Pulmonary Fibrosis Through Anti-Inflammatory and Anti-Fibrotic Process in Rodent Pulmonary Fibrosis Model and Cellular Epithelial–Mesenchymal Transition Model
Source: Molecules. 2025 Apr 18;30(8):1821. doi: 10.3390/molecules30081821 (PMC12029700; doi:10.3390/molecules30081821)
Supplement: Supplementary file 1 [file molecules-30-01821-s001.zip › Western Blot original images-GAPDH.pptx]

## Slide 1
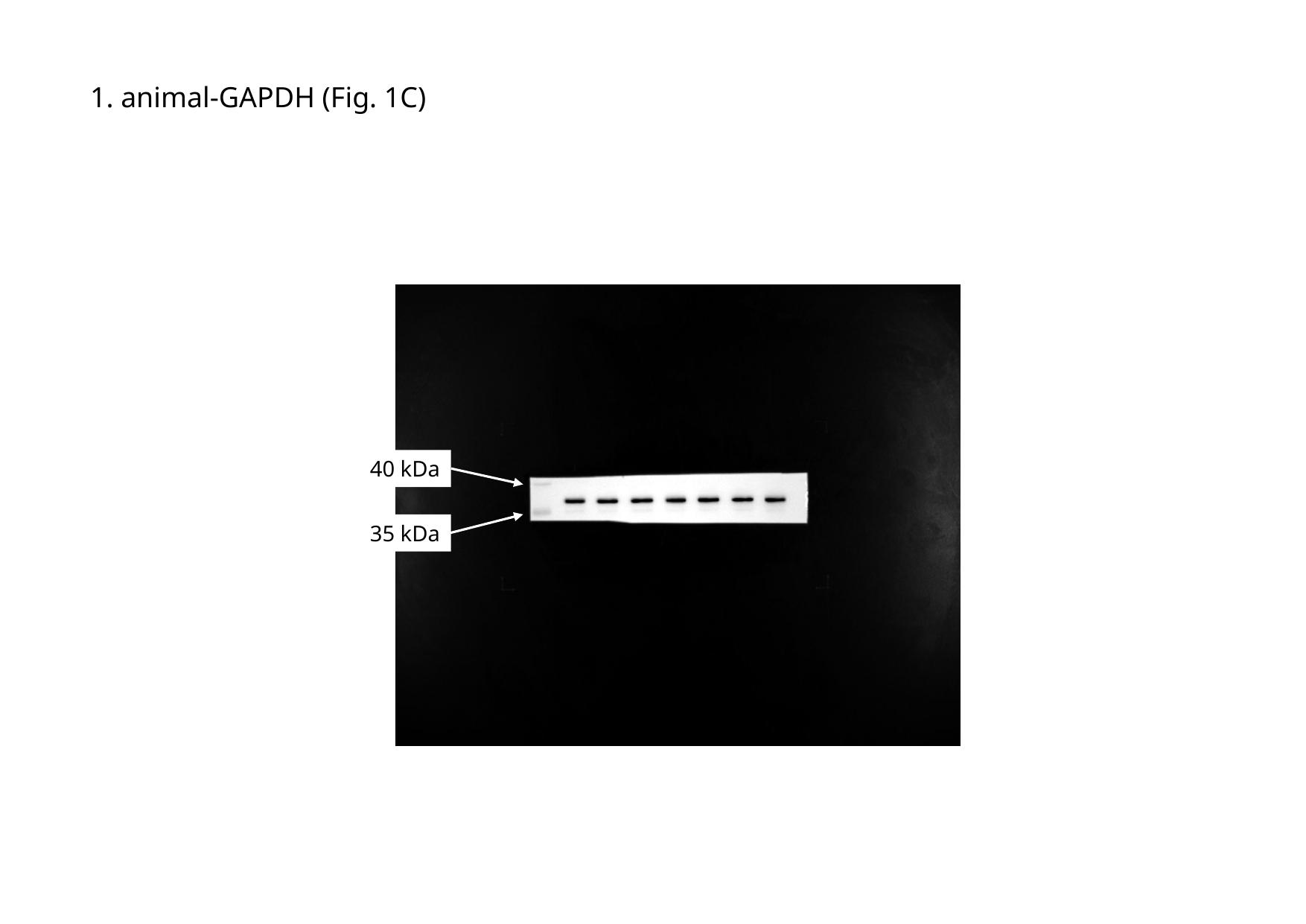

1. animal-GAPDH (Fig. 1C)
40 kDa
35 kDa

## Slide 2
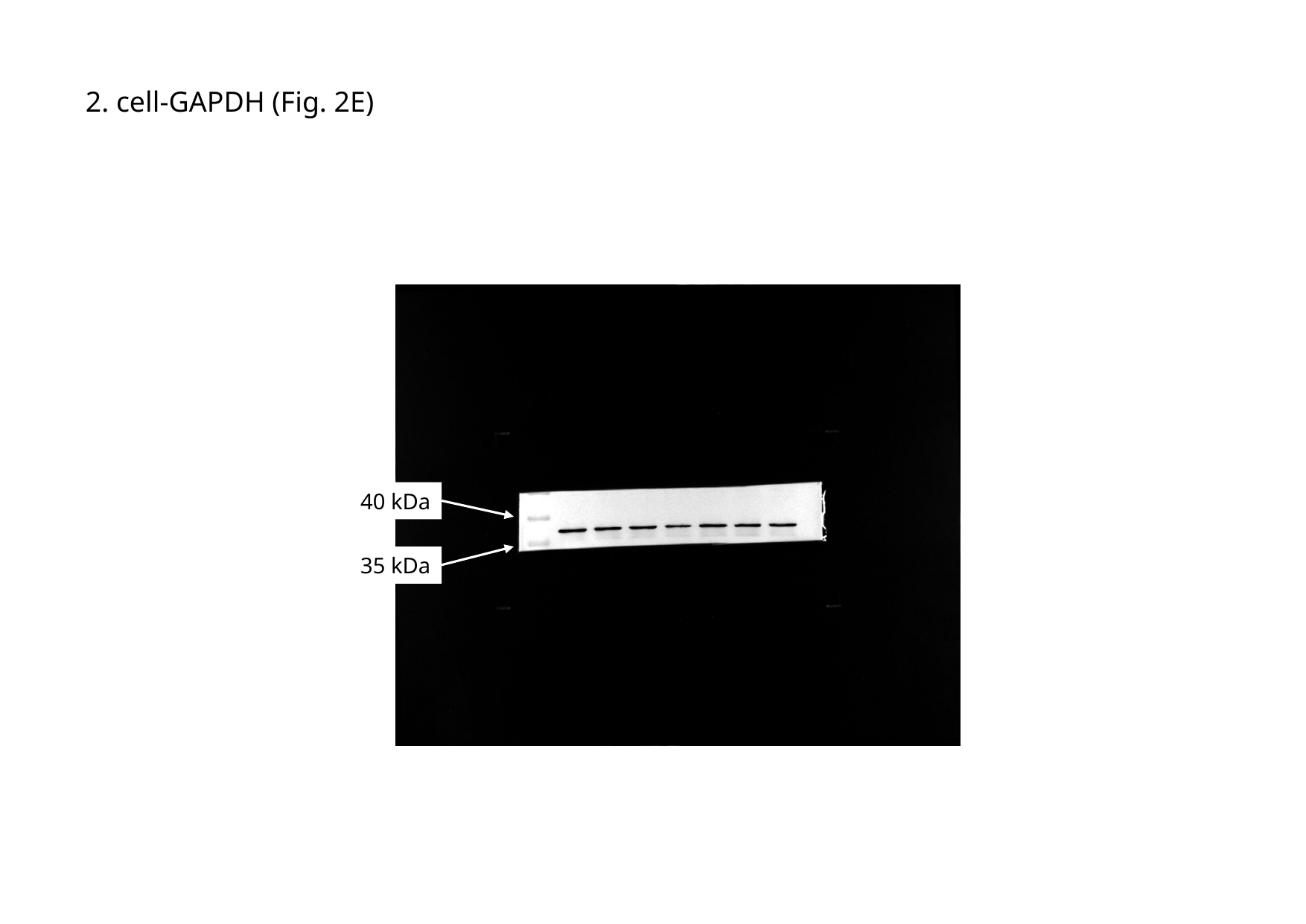

2. cell-GAPDH (Fig. 2E)
40 kDa
35 kDa

## Slide 3
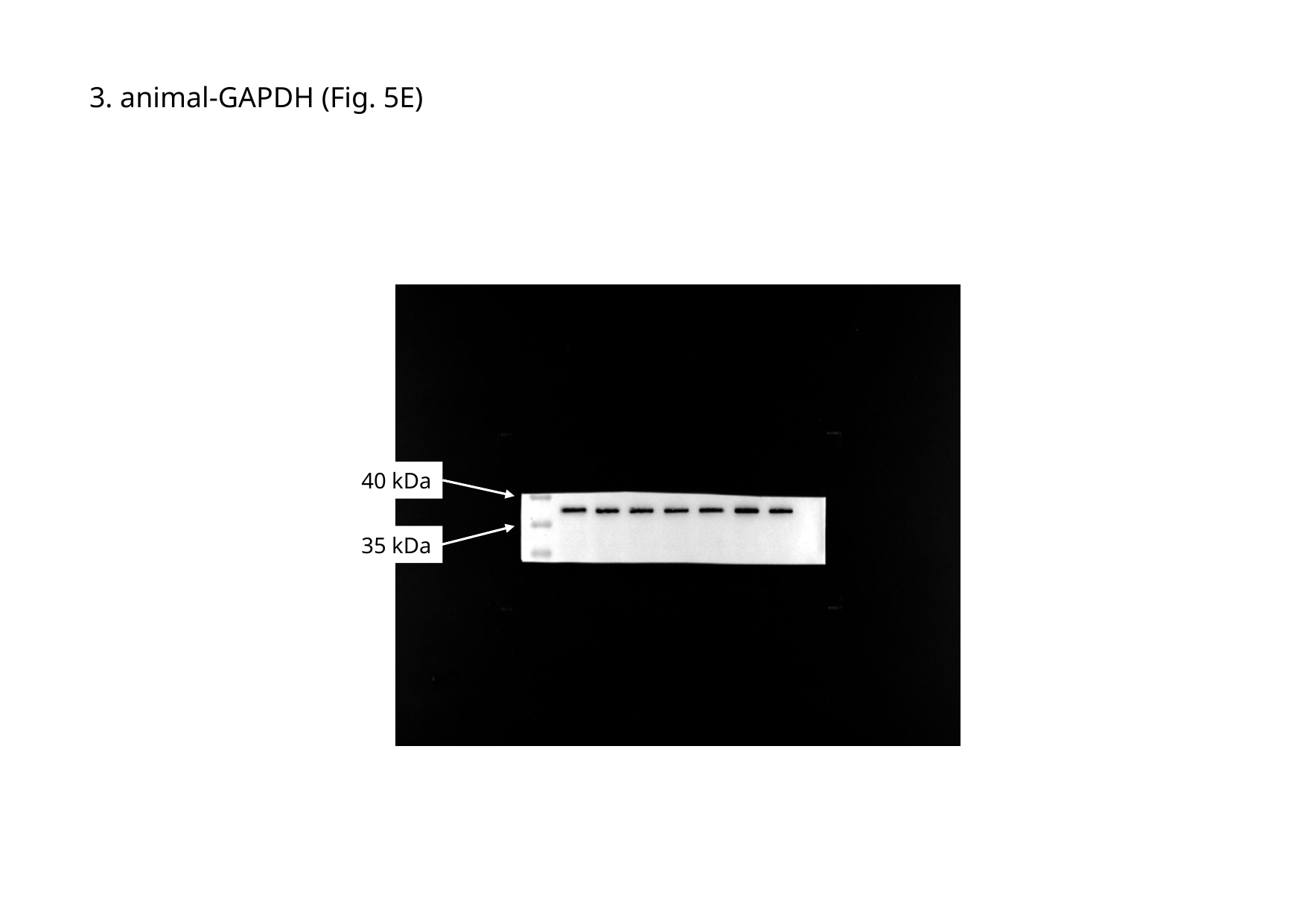

3. animal-GAPDH (Fig. 5E)
40 kDa
35 kDa

## Slide 4
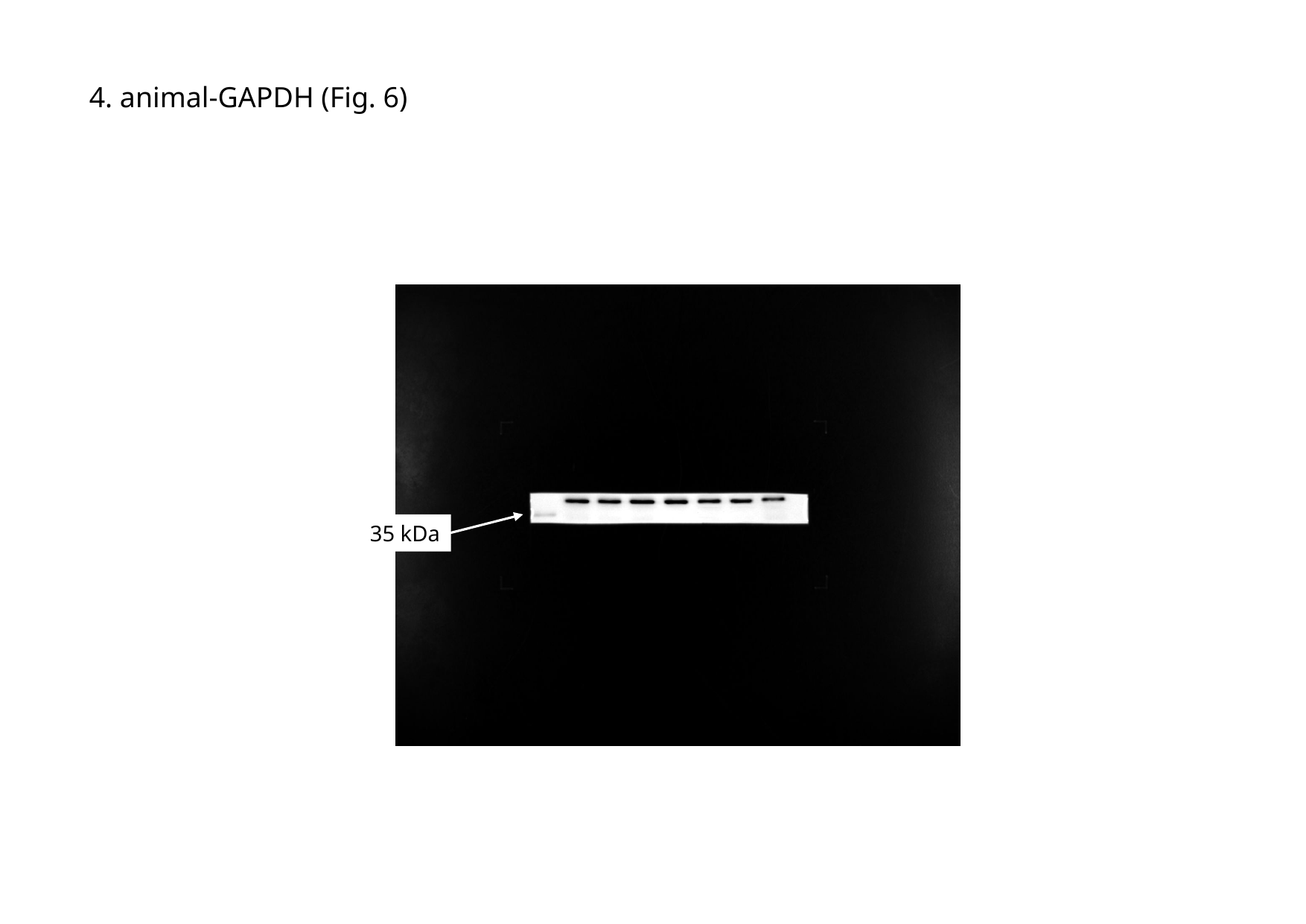

4. animal-GAPDH (Fig. 6)
35 kDa

## Slide 5
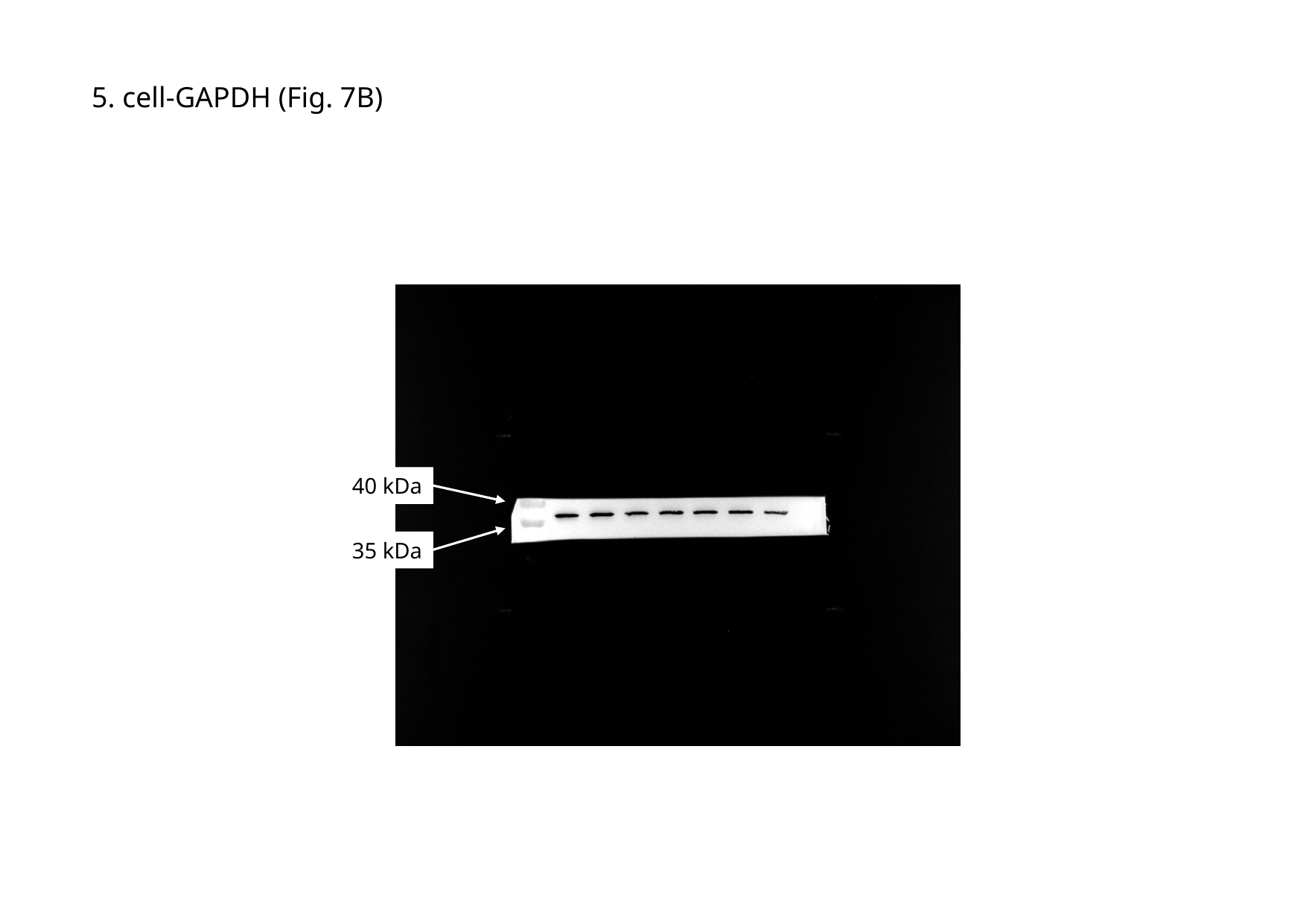

5. cell-GAPDH (Fig. 7B)
40 kDa
35 kDa
